# Successful isolation of *Leishmania infantum* from *Rhipicephalus sanguineus* sensu lato (Acari: Ixodidae) collected from naturally infected dogs

**DOI:** 10.1186/s12917-015-0576-5

**Published:** 2015-10-09

**Authors:** Viviane Medeiros-Silva, Rodrigo Gurgel-Gonçalves, Nadjar Nitz, Lucia Emilia D’ Anduraim Morales, Laurício Monteiro Cruz, Isabele Gonçalves Sobral, Mariana Côrtes Boité, Gabriel Eduardo Melim Ferreira, Elisa Cupolillo, Gustavo Adolfo Sierra Romero

**Affiliations:** Programa de Pós-Graduação em Medicina Tropical, Universidade de Brasília, Brasília, DF Brazil; Laboratório de Parasitologia Médica e Biologia de Vetores, Universidade de Brasília, Brasília, DF Brazil; Diretoria de Vigilância Ambiental do Distrito Federal, Brasília, DF Brazil; Laboratório de Pesquisa em Leishmaniose, Coleção de Leishmania, Instituto Oswaldo Cruz, Fundação Oswaldo Cruz, Rio de Janeiro, RJ Brazil; Laboratório de Leishmanioses, Núcleo de Medicina Tropical, Universidade de Brasília, Brasília, DF Brazil

**Keywords:** *Leishmania infantum*, *Rhipicephalus sanguineus*, PCR, *Canis familiaris*

## Abstract

**Background:**

The main transmission route of *Leishmania infantum* is through the bites of sand flies. However, alternative mechanisms are being investigated, such as through the bites of ticks, which could have epidemiological relevance. The objective of this work was to verify the presence of *Leishmania* spp. in *Rhipicephalus sanguineus* sensu lato collected from naturally infected dogs in the Federal District of Brazil.

**Methods:**

Ticks were dissected to remove their intestines and salivary glands for DNA extraction and the subsequent amplification of the conserved region of 120 bp of kDNA and 234 bp of the hsp70 gene of *Leishmania* spp. The amplified kDNA products were digested with endonucleases HaeIII and BstUI and were submitted to DNA sequencing. Isolated *Leishmania* parasites from these ticks were analyzed by multilocus enzyme electrophoresis, and the DNA obtained from this culture was subjected to microsatellite analyses.

**Results:**

Overall, 130 specimens of *R. sanguineus* were collected from 27 dogs. *Leishmania* spp. were successfully isolated in culture from five pools of salivary glands and the intestines of ticks collected from four dogs. The amplified kDNA products from the dog blood samples and from the tick cultures, when digested by HaeIII and BstUI, revealed the presence of *L. braziliensis* and *L. infantum.* One strain was cultivated and characterized as *L. infantum* by enzyme electrophoresis. The amplified kDNA products from the blood of one dog showed a sequence homology with *L. braziliensis*; however*,* the amplified kDNA from the ticks collected from this dog showed a sequence homology to *L. infantum.*

**Conclusion:**

The results confirm that the specimens of *R. sanguineus* that feed on dogs naturally infected by *L. infantum* contain the parasite DNA in their intestines and salivary glands, and viable *L. infantum* can be successfully isolated from these ectoparasites.

**Electronic supplementary material:**

The online version of this article (doi:10.1186/s12917-015-0576-5) contains supplementary material, which is available to authorized users.

## Background

The main transmission route of *Leishmania infantum* (syn. *Leishmania chagasi*) is through the bite of sand flies (Diptera: Psychodidae). In Brazil, the parasite is primarily transmitted by *Lutzomyia longipalpis* [1]. However, alternative transmission modes are being investigated [2–7], and transmission of *Leishmania* spp. through bites from or through the ingestion of infected fleas and ticks could be epidemiologically relevant in domestic dogs [8–10].

Some evidence suggests the possibility that the dog tick *Rhipicephalus sanguineus* sensu lato (Acari: Ixodidae) could act as either a biological [11] or mechanical vector of *L. infantum* [8, 12, 13]*.* In addition, molecular studies indicate the possibility of transovarial transmission of *L. infantum* in *R. sanguineus* [13, 14]. These results highlight the potential of *R. sanguineus* as a vector of *L. infantum.* However, one problem that remains unresolved is why *L. infantum* DNA is present in ticks that feed on infected animals when, so far, it has not been possible to isolate viable parasites from *R. sanguineus* who fed on naturally infected dogs.

The Federal District of Brazil (FD) has recently become an endemic area for human and canine visceral leishmaniasis [15], where there are many infected dogs and the presence of *Lu. longipalpis* [16]. This scenario is appropriate for investigating the infection of *R. sanguineus* by *Leishmania* spp. and for evaluating the potential role of *R. sanguineus* in the transmission of canine visceral leishmaniasis (CVL). Thus, the aim of this study was to verify the presence of *Leishmania* spp. in *R. sanguineus* sensu lato collected from naturally infected dogs in the FD. Moreover, we targeted the isolates to identify the species of *Leishmania* in *R. sanguineus* and to determine their location in the ectoparasites.

## Methods

### Canine samples

This study included domestic dogs collected by the Diretoria de Vigilância Ambiental (DIVAL) of the FD during the survey of the CVL control program in the region. Dogs were either voluntarily supplied by their owners to DIVAL officers during the CVL survey, or stray dogs collected by routine DIVAL activities in the FD were included in the study. For convenience, the sample consisted of dogs collected in the period ranging from 4 July to 30 September in 2011 after approval was granted by the Animal Ethics Committee of the Faculty of Medicine at University of Brasilia (Process No. 57444/2011). To be included in the study, dogs must have presented at least one positive result after three serological tests used for detection of *Leishmania* spp. infection (see below). Dogs vaccinated against leishmaniasis, under suspicion of and/or confirmed with rabies and those using collars impregnated with insecticides were excluded from this study.

Blood samples from each dog (3 mL) were collected by venipuncture of the cephalic, femoral or jugular vein, and transferred to tubes with an anticoagulant (Vacuette® K3E K_3_ EDTA). Two tubes were collected from each dog: one to undergo serologic testing by enzyme-linked immunosorbent assay (ELISA) and indirect immunofluorescence antibody test (IFAT) and the other to undergo DNA extraction and the rapid immunoassay dual-path platform test (DPP). The plasma samples were frozen at −20 °C until testing.

### Tick samples

A clinical examination was performed in anesthetized dogs, with preferred places, such as the ears, interdigital spaces, and the areas around the orbits, being explored for ticks. The ticks were collected with tweezers, stored in properly identified tubes and kept at room temperature for approximately 2 h until the identification and dissection procedure. The ectoparasites were separated by sex and morphologically identified prior to their dissection based on taxonomic keys [17, 18].

The dissection was performed while the ticks were still alive according to the procedure described by Edwards et al. [19] After the internal contents were exposed, the salivary glands (SGs) were removed and then the intestine. The SGs and intestine samples were placed in separate Eppendorf tubes containing 0.2 mL of 0.9 % saline and 5-fluorocytosine (100 μg/mL) and stored at −20 °C for later DNA extraction.

**Fig. 1:**
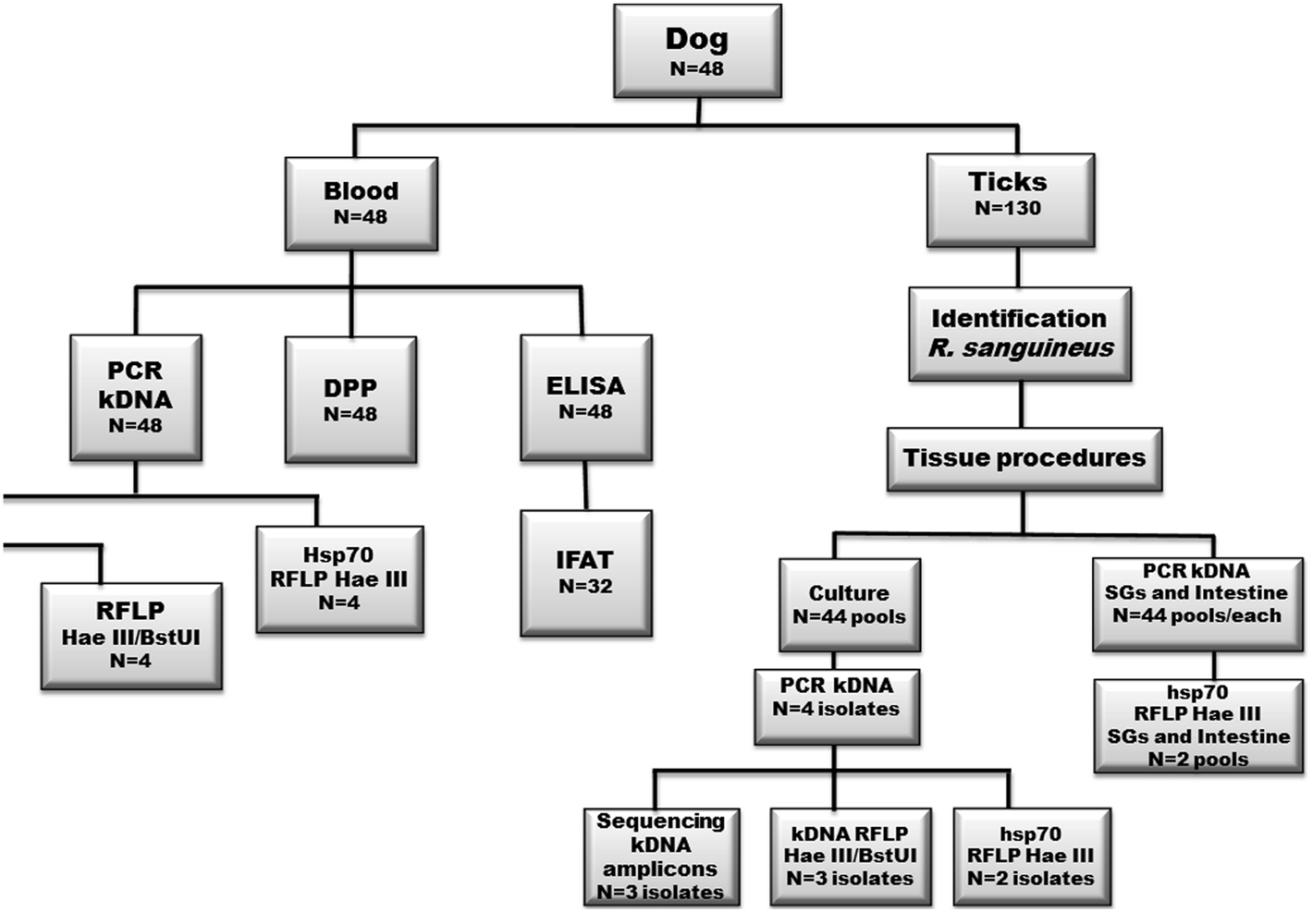
Sampling processing flow. SG: salivary gland; Int: intestine; Seq: sequencing; Ø: nymph. RFLP: restriction fragment lengh polymorphism. The numbers in the boxes show the number of samples/pools processed

The ticks taken from each dog were separated into pools (of up to 10 ticks) of males, females and nymphs. The processing flow of these samples is shown in Fig. 1. After isolation of the salivary glands and of the intestine of each tick, the remaining extravasated content was transferred to an Eppendorf tube containing 0.2 mL of 0.9 % saline and 5-fluorocytosine (100 μg/mL) and kept cool at 8 °C for 30 min to one hour. Then, the material was inoculated into the culture media [20]. These cultures were evaluated every 2 days until the 30th day.

### Serological diagnosis

The blood samples intended for the ELISA (EIE-LVC kit Bio-Manguinhos/FIOCRUZ, Rio de Janeiro, Brazil) and the IFAT (IFI-LVC kit Bio-Manguinhos/FIOCRUZ, Rio de Janeiro, Brazil) tests were stored and processed by DIVAL. Only samples with positive ELISA results were subjected to IFAT for confirmation of positivity. The IFAT cut-off was 1:40.

The rapid immunochromatographic test, DPP® Canine Visceral Leishmaniasis (Bio-Manguinhos, Brazil), was performed according to the manufacturer’s instructions on plasma samples stored at −20 °C that had been thawed at room temperature.

### DNA extraction and PCR

DNA obtained from blood samples was extracted using a Wizard Genomic DNA Purification Kit (Promega, Madison, WI, USA). Salivary glands and intestine samples were processed using Illustra Tissue Cells & Genomic Prep Mini Spin kit (GE Healthcare, New York, USA). DNA extraction of the parasites isolated in the culture medium was performed by the phenol-chloroform technique. Primers used to amplify 120 bp of the conserved kDNA region of *Leishmania* spp. were as follows: BW-B: 5 'CCG CCC CTA TTT TAC CCC ACC ACC 3'; FW: 5 'GGG GAG GGG CGT TCT GCG AA 3'; BW-CA: 5 'GGC CCA CTA TAT TAC ACC AAC CCC 3' [15]. The amplification conditions included a final volume of 10 μL, with the following standard mixture for each reaction: 1 μL Buffer (5x), 1 μL dNTPs (2 mM), 1 μL MgCl_2_ (25 mM), 1 μL FW primer (1.2 mM), 1 μL BW-B/BW-CA primer (0.6 mM), 0.1 μL Taq DNA polymerase (5 μ/μL), 3.9 μL of H_2_O and 1 μL of DNA template. The thermocycling conditions were 95 °C for 5 min for denaturation; then 39 cycles of 30 s at each temperature of 95 °C, 66 °C and 72 °C; and 5 min of final extension at 72 °C.

The primers used to amplify a 234-bp target of hsp70 of *Leishmania* spp. were FW: 5 'GAT CGA CGA GGA GGC CAT GGT 3' and BW: 5 'GAC TTC TCC GCC TCC TGG TTG 3'. The amplification yielded a final volume of 25 μL, and the standard mix for each reaction was as follows: 5 μL Buffer (5x), 2 μL of dNTPs (25 mM), 1.5 μL MgCl_2_ (25 mM) 0.25 μL FW primer (20 pm), 0.25 BW μL of primer (20 pm), 0.15 μL of Taq DNA polymerase (5 μ/μL), 14.85 μL of H_2_O and 1 μL of DNA. The thermocycling conditions were: 94 °C for 5 min for denaturation; 30 cycles of 94 °C for 30 s, 63 °C for 1 min and 72 °C for 10 min; and final extension at 72 °C for 10 min.

All PCR products were examined by electrophoresis in a polyacrylamide gel at 7.5 %, 150 V, and 75 Amp for 90 min. The molecular weight marker used in all gels was the DNA Molecular Weight Marker V (Roche Applied Science, Germany).

### Enzymatic digestion with restriction endonucleases

The PCR-RFLP test with the HaeIII restriction enzyme required 5 μL of PCR products plus enzyme, which were incubated in a water bath at 37 °C. BstUI digestion was performed with 10 μL of PCR products plus enzyme, which were incubated in a water bath at 60 °C. The sample digested by the HaeIII and the sample digested by the BstUI enzymes were allowed to incubate for 1 h, and then they were subjected to electrophoresis in a polyacrylamide gel at 7.5 %.

### DNA sequencing

PCR products were purified with an Illustra GFX PCR DNA & Gel Band Purification Kit (GE Healthcare, New York, USA), according to the manufacturer’s instructions. Sequencing was performed by the company Genomic Engenharia Molecular with the BigDye® Terminator v3.1 Cycle Sequencing Kit from Applied Biosystems. Obtained sequences were edited by the DNAMAN software (Lynnon Corporation, Canada). Later on, these sequences were compared with the sequences of *Leishmania* species available in GenBank through the BLASTn algorithm (Basic Local Alignment Search Tool) from the National Center for Biotechnology Information of the United States of America.

### Multilocus analysis (MLEE - multilocus enzyme electrophoresis and MLMT – multilocus microsatellite typing)

The *Leishmania* culture was maintained on a semi-solid medium and is on deposit in the *Leishmania* Collection at the Oswaldo Cruz Institute. This culture was typed by MLEE, as described [21]. The DNA obtained from this culture were subjected to microsatellite analysis, using a previously described protocol [22]. The MLEE profiles obtained were compared with a reference strain for *L. infantum* (MHOM/BR/1974/PP75). The microsatellite data were compared with the panel presented in Ferreira et al. [22].

## Results

Overall, 48 dogs (23 males and 25 females) were included. Fourteen were of unknown age, six were between 5 months and one year, 19 were between 1 and 7 years, and nine were between 8 and 14 years old.

Twenty-seven dogs (56.2 %) were parasitized by *R. sanguineus* senso lato (*n* = 130 ticks: 61 males, 7 females and 62 nymphs).

Thirty-two dogs (66.7 %) were positive in the ELISA test for leishmaniasis. These positive samples were subjected to the IFAT, resulting in 81.2 % positivity (26/32). The DPP test was positive in 56.2 % of the 48 tested dogs. Of the 48 dogs, 34 (70.8 %) had at least one of the three tests showing a positive or reactive result. The 120-bp kDNA PCR was positive in blood samples from 26 dogs (54.2 %).

Five of the 44 culture tubes inoculated with tick pools collected from four dogs had growth of flagellated forms compatible with *Leishmania* spp. Two of them were pools of male ticks, and three, pools of female ticks.

The 120-bp kDNA PCRs of the intestine and SGs pools of *R. sanguineus* sensu lato were positive in 25 samples obtained from 10 dogs (Table 1). The results of all the methods applied to both canine and ticks samples that showed growth of *Leishmania* spp. are demonstrated in Table 2, and the complete data are detailed in the Additional file 1: Table S1.

**Table 1:** Results of kDNA detection by PCR in pools of salivary glands and intestines of *Rhipicephalus sanguineus* sensu lato collected from dogs in an endemic area of visceral leishmaniasis in the Federal District, Brazil, 2011

	kDNA PCR	
Samples	Positive/Examined pools	Positivity (%)
Salivary glands (*n* = 44)		
Females	7/21	33.3
Males	5/20	25.0
Nymphs	1/3	33.3
Subtotal	13/44	29.5
Intestines (*n* = 44)		
Females	6/21	28,6
Males	5/20	25.0
Nymphs	1/3	33.3
Subtotal	12/44	27.3
Total	25/88	28,4

**Table 2:** Results of all methods applied to the samples taken from dogs and ticks that showed growth of a *Leishmania* culture from the material obtained from the ticks, Federal District, Brazil, 2011

Dogs						Ticks				Cultures		
Dog	ELISA	IFI	DPP	PCR Canine blood (kDNA)	PCR Canine blood (hsp70)	Collected ticks	Tick sex (n)	PCR (kDNA) Pool SG	PCR (kDNA) Pool Int.	Culture (NNN)	PCR (kDNA) Culture	PCR (hsp70) Culture
I	Reagent	1/80	Positive	Positive	Negative	6	Male (1)	Positivo	Negative	Negative	ND	ND
							Female (5)	Positive	Positive	Positive	Positive	Positive
II	Reagent	1/80	Positive	Positive	Negative	5	Male (5)	Positive	Positive	Positive	Negative	Negative
III	Reagent	1/80	Positive	Positive	Positive	8	Male (5)	Positive	Positive	Positive	Positive	Positive
							Female (3)	Positive	Positive	Positive	Positive	Positive
IV	Reagente	1/40	Positive	Positive	Positive	2	Male (1)	Negative	Negative	Negative	ND	ND
							Female (1)	Positive	Positive	Positive	Positive	Positive

ND: not done; SG: Salivary glands; Int.: Intestines

**Fig. 2:**
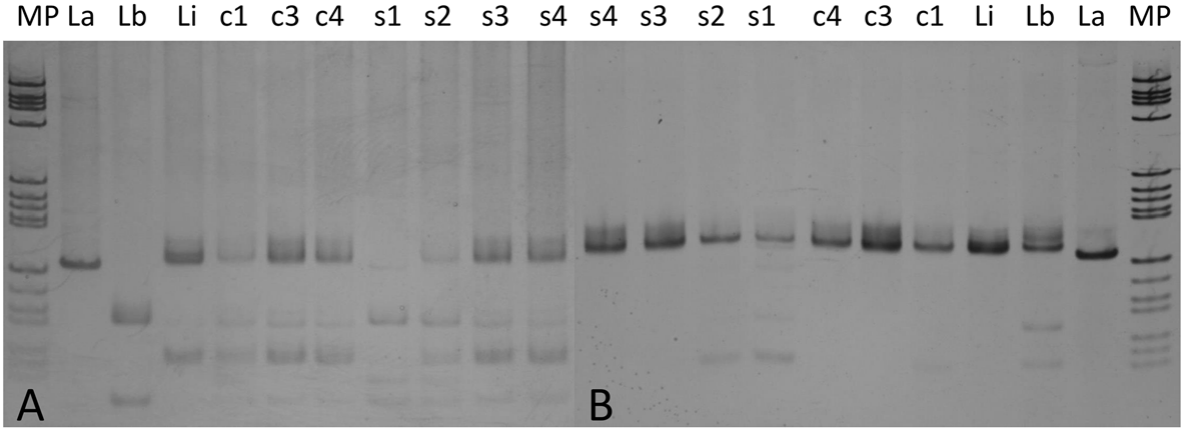
Amplified 120pb kDNA products digested by the enzymes Hae III (**a**) and BstUI (**b**) in canine blood samples and cultures from the tick contents. Reference strain cultures of *L. amazonensis* (La), *L. braziliensis* (Lb), and *L. infantum* (Li); Cultures from *R. sanguineus* specimens obtained from dog I (c1), dog III (C3), and dog IV (c4); Blood samples of dog I (s1), dog II (s2), dog III (s3), dog IV (4); MP: molecular weight marker

PCR kDNA amplicons from blood samples obtained from dogs I and II, when digested by the enzymes HaeIII and BstUI, were similar to the *L. (V.) braziliensis* profile (Fig. 2). In addition, the PCR kDNA amplicons from the culture obtained from the ticks collected from dog I and digested by BstUI, were compatible with *L. (V.) braziliensis.* However, the kDNA amplicons from the blood samples and tick cultures obtained from dogs III and IV, when digested by the same enzymes, showed a profile similar to *L. infantum* (Fig. 2).

The sequence of kDNA amplicons from the blood of dog I share 94 % identity with a sequence of *Leishmania* (*V*.) (X54472.1), a result consistent with the results of the kDNA digestion by HaeIII and BstUI*.* The sequence of the product resulting from the culture of the SGs and intestine of ticks from this dog was identified as *L. infantum.* The sequences of the amplified products from the blood samples of dogs II, III and IV showed 90 to 97 % identity with sequences of *L. infantum* (EU437406.1 and EU370885.1). The same pattern of identity was observed for amplicons from cultures of the SGs and intestines of ticks from dogs III and IV.

Heat shock protein 70 PCR coupled with HaeIII RFLP was performed in a limited number of samples: four dog blood samples, two culture isolates and two pools of SGs and intestine; all the samples belonged to the group of four kDNA-positive dogs whose ticks yielded positive parasite cultures. The RFLP profile obtained with amplicons of the hsp70 PCR digested by HaeIII were consistent with the species identity obtained by the other methods.

The isolated strain from dog III (ARHI/BR/2011/NMT-DF-2595) showed an isoenzyme profile compatible with *L. infantum* zymodeme 1 [21]. The allele profile analysis obtained by MLMT showed that this strain belongs to POP1, the population of *L. infantum* most prevalent and most dispersed in Brazil [22].

## Discussion

The results of this study show that, under natural conditions, *R. sanguineus* specimens that feed on dogs naturally infected with *L. infantum* contain the parasite DNA in their intestine and salivary glands. Furthermore, the study confirmed the presence of viable flagellated forms compatible with *L. infantum* in the *R. sanguineus* obtained from these dogs. The successful isolation of *L. infantum* by inoculating samples of *R. sanguineus* in a culture medium is further evidence of the potential of this tick for the transmission of CVL.

Although extremely laborious and delicate, the dissection of ticks is a process that allows evaluation of the internal contents (salivary glands and intestines) of these ectoparasites, allowing specific molecular evaluations. Some studies that have adopted this approach, either detecting DNA of *L. infantum* in the salivary glands of the ticks collected from naturally infected dogs [10, 14, 23] or by using macerated ticks to perform PCR [8], have also succeeded in detecting the DNA of *L. infantum*, which is not sufficient to prove the viability of the parasite inside the ticks.

There are reports of the successful isolation of *Leishmania* only in ticks that fed experimentally on dogs naturally infected by *L. infantum* [11, 13], and to date, most of the evidence is limited to the detection of parasite DNA in the specimens studied. In the present study, isolation was possible from pools of unfed male ticks, suggesting that *Leishmania* could persist in *R. sanguineus* after blood digestion. However, further studies detecting the *Leishmania* morphological stages and determining the parasite load by quantifying the DNA (qPCR) in ticks collected from dogs and analyzed from different periods may further clarify for how long the different forms of *Leishmania* can remain in the tick after digestion of the blood.

Based on previous comparative studies [24], kDNA PCR was expected to be the most sensitive genus-specific molecular technique, which may explain the positive rates of kDNA amplification from the DNA extracted from the blood samples of the four dogs and the two negative samples in the hsp70 assay, which is less sensitive.

The kDNA PCR-amplified DNA from the blood samples of the dogs and of the parasite cultures isolated from the ticks were subjected to sequencing, which allowed the definitive identification of the detected trypanosomatids. This method was adopted since we cannot rule out the occurrence of monoxenic trypanosomatids in ticks and because positive PCR results for this target are not sufficient to indicate a *Leishmania* infection [25].

The identification by MLEE of the strain that was isolated and successfully cultured indicated the presence of the *L. infantum* zymodeme 1 [21], in agreement with previous studies that indicated this is the major circulating zymodeme in Brazil [21, 22]. The MLMT analyses show that this strain belongs to the POP1 classification, following the analysis performed by Ferreira et al. [22]. Strains of *L. infantum* belonging to POP1 have been described in the FD and have been isolated from both dogs and humans [22].

The results of the sequencing and digestion by HaeIII and BstUI demonstrated the presence of *L. braziliensis* and *L. infantum* in samples from dog I, suggesting a co-infection as has already been shown in other studies [26–28].

The visualization of the developmental stages of *Leishmania* spp. in the intestines and salivary glands of ticks would contribute to a better understanding of the cycle of the parasite inside the ectoparasite. For this, another chronological method for dissecting the ticks should be adopted, first using the internal content for the preparation of slides in an attempt to find more parasites, and subsequent analysis by PCR and culture. Finally, experiments similar to those made by Mackenzie [11], who attempted to reproduce the parasite cycle under experimental conditions by feeding ticks on infected dogs and observing *Leishmania* development inside the tick intestine, should be repeated and enhanced in an attempt to obtain consistent results, especially directed to prove *R. sanguineus* sensu lato vectorial competence and capacity.

## Conclusions

Our results indicate that the specimens of *R. sanguineus* that feed on dogs naturally infected by *L. infantum* contain parasite DNA in their intestines and salivary glands. Additionally, we demonstrated that it is possible to isolate viable *L. infantum* from this ectoparasite. However, our findings are not enough to confirm or presume that ticks are potential vectors of *L. infantum*. Further studies should be conducted to better understand the relevance of these findings to the potential competence of *R. sanguineus* for the maintenance of visceral leishmaniasis transmission.

## Additional file

Additional file 1: Table S1. Complete data of diagnostic tests applied to samples from 48 dogs and ticks collected from them. Brasilia, Federal District. Brazil. (DOCX 33 kb)

## Abbreviations

CVL: Canine visceral leishmaniasis
DIVAL: Diretoria de Vigilância Ambiental (Enviromental Surveillance Department)
DPP: Dual-path platform test
ELISA: Enzyme-linked immunosorbent assay
FD: Federal District of Brazil
IFAT: Indirect immunofluorescence antibody test
MLEE: Multi-Locus Enzyme Electrophoresis
MLMT: Multi-Locus Microsatellite Typing
SGs: Salivary glands
RFLP: Restriction fragment lengh polymorphism
