# Supplementary material for: Successful isolation of Leishmania infantum from Rhipicephalus sanguineus sensu lato (Acari: Ixodidae) collected from naturally infected dogs
Source: BMC Vet Res. 2015 Oct 9;11:258. doi: 10.1186/s12917-015-0576-5 (PMC4600268; doi:10.1186/s12917-015-0576-5)
Supplement: Additional file 1: Table S1. — Complete data of diagnostic tests applied to samples from 48 dogs and ticks collected from them. Brasilia, Federal District. Brazil. (DOCX 33 kb) [file 12917_2015_576_MOESM1_ESM.docx]

**Additional file 1: Table S1. Complete data of diagnostic tests applied to samples from 48 dogs and ticks collected from them. Brasilia, Federal District. Brazil.**

| **Dog** | **ELISA** | **IFI** | **DPP** | **PCR (kDNA) Canine blood** | **Collected**  **Ticks (*n*)** | **Number of ticks by sex and stage** | | | **PCR (kDNA)**  **Pools of salivary glands**  **(*n* =44 *pools*)** | | | **PCR (kDNA)**  **Pools of Intestines**  **(*n* =44 *pools*)** | | | **Cultures** |
| --- | --- | --- | --- | --- | --- | --- | --- | --- | --- | --- | --- | --- | --- | --- | --- |
|  |  |  |  |  |  | **Female** | **Male** | **Nymph** | **Female** | **Male** | **Nymph** | **Female** | **Male** | **Nymph** |  |
| **01** | Reactive | 1/80 | Positive | Positive | 01 | 01 |  |  | Negative |  |  | Negative |  |  |  |
| **02** | NR | ND | Positive | Negative | 06 | 04 | 02 |  | Negative | Negative |  | Negative | Negative |  |  |
| **03** | NR | ND | Negative | Negative | 05 |  | 05 |  |  | Negative |  |  | Negative |  |  |
| **04** | Reactive | 1/80 | Positive | Positive | 06 | 05 | 01 |  | Positive | Positive |  | Positive | Negative |  | Positive (F) |
| **05** | Reactive | 1/80 | Positive | Positive | 07 | 05 | 02 |  | Positive | Negative |  | Positive | Positive |  |  |
| **06** | NR | ND | Negative | Negative | 05 | 02 | 03 |  | Negative | Negative |  | Negative | Negative |  |  |
| **07** | NR | ND | Negative | Negative | 0 |  |  |  |  |  |  |  |  |  |  |
| **08** | NR | ND | Negative | Negative | 0 |  |  |  |  |  |  |  |  |  |  |
| **09** | NR | ND | Negative | Negative | 0 |  |  |  |  |  |  |  |  |  |  |
| **10** | Reactive | 1/40 | Positive | Positive | 0 |  |  |  |  |  |  |  |  |  |  |
| **11** | Reactive | ND | Negative | Positive | 01 |  | 01 |  |  | Negative |  |  | Negative |  |  |
| **12** | NR | ND | Positive | Negative | 02 | 01 | 01 |  | Negative | Negative |  | Negative | Negative |  |  |
| **13** | NR | ND | Negative | Negative | 0 |  |  |  |  |  |  |  |  |  |  |
| **14** | NR | ND | Negative | Negative | 0 |  |  |  |  |  |  |  |  |  |  |
| **15** | Reactive | ND | Negative | Negative | 03 |  |  | 03 |  |  | Positive |  |  | Positive |  |
| **16** | Reactive | 1/80 | Positive | Positive | 06 | 04 | 02 |  | Positive | Positive |  | Positive | Positive |  |  |
| **17** | Reactive | ND | Negative | Negative | 10 | 05 | 05 |  | Negative | Negative |  | Negative | Negative |  |  |
| **18** | Reactive | 1/80 | Positive | Positive | 0 |  |  |  |  |  |  |  |  |  |  |
| **19** | Reactive | ND | Positive | Positive | 0 |  |  |  |  |  |  |  |  |  |  |
| **20** | Reactive | 1/80 | Positive | Positive | 0 |  |  |  |  |  |  |  |  |  |  |
| **21** | Reactive | 1/40 | Positive | Positive | 0 |  |  |  |  |  |  |  |  |  |  |
| **22** | NR | ND | Negative | Negative | 03 |  | 03 |  |  | Negative |  |  | Negative |  |  |
| **23** | Reactive | 1/80 | Positive | Positive | 06 | 04 | 02 |  | Positive | Positive |  | Negative | Negative |  |  |
| **24** | Reactive | 1/80 | Positive | Positive | 05 | 03 |  | 02 | Negative |  | Negative | Negative |  | Negative |  |
| **25** | NR | ND | Negative | Negative | 06 |  | 06 |  |  | Negative |  |  | Negative |  |  |
| **26** | Reactive | ND | Negative | Negative | 06 | 06 |  |  | Negative |  |  | Negative |  |  |  |
| **27** | Reactive | 1/80 | Positive | Negative | 04 | 02 |  | 02 | Negative |  | Negative | Negative |  | Negative |  |
| **28** | Reactive | 1/80 | Positive | Positive | 05 |  | 05 |  |  | Positive |  |  | Positive |  | Positive (M) |
| **29** | Reactive | 1/80 | Positive | Positive | 0 |  |  |  |  |  |  |  |  |  |  |
| **30** | Reactive | 1/80 | Negative | Positive | 0 |  |  |  |  |  |  |  |  |  |  |
| **31** | Reactive | 1/80 | Positive | Positive | 0 |  |  |  |  |  |  |  |  |  |  |
| **32** | Reactive | 1/80 | Positive | Negative | 0 |  |  |  |  |  |  |  |  |  |  |
| **33** | Reactive | 1/80 | Positive | Negative | 02 | 02 |  |  | Negative |  |  | Negative |  |  |  |
| **34** | NR | ND | Negative | Positive | 15 | 05 | 10 |  | Negative | Negative |  | Negative | Negative |  |  |
| **35** | Reactive | 1/80 | Positive | Positive | 0 |  |  |  |  |  |  |  |  |  |  |
| **36** | Reactive | 1/80 | Positive | Positive | 08 | 03 | 05 |  | Positive | Positive |  | Positive | Positive |  | Positive (F and M) |
| **37** | Reactive | 1/80 | Positive | Positive | 0 |  |  |  |  |  |  |  |  |  |  |
| **38** | Reactive | 1/80 | Positive | Positive | 01 | 01 |  |  | Positive |  |  | Positive |  |  |  |
| **39** | NR | ND | Negative | Negative | 03 | 02 | 01 |  | Negative | Negative |  | Negative | Negative |  |  |
| **40** | NR | ND | Negative | Positive | 04 | 02 | 02 |  | Negative | Negative |  | Negative | Negative |  |  |
| **41** | NR | ND | Negative | Negative | 0 |  |  |  |  |  |  |  |  |  |  |
| **42** | Reactive | 1/80 | Positive | Positive | 0 |  |  |  |  |  |  |  |  |  |  |
| **43** | Reactive | 1/40 | Positive | Negative | 0 |  |  |  |  |  |  |  |  |  |  |
| **44** | Reactive | 1/40 | Positive | Positive | 02 | 01 | 01 |  | Positive | Negative |  | Positive | Negative |  | Positive (F) |
| **45** | NR | ND | Negative | Negative | 0 |  |  |  |  |  |  |  |  |  |  |
| **46** | Reactive | ND | Negative | Negative | 04 | 03 | 01 |  | Negative | Negative |  | Negative | Negative |  |  |
| **47** | Reactive | 1/80 | Negative | Positive | 0 |  |  |  |  |  |  |  |  |  |  |
| **48** | Reactive | 1/80 | Positive | Positive | 04 | 01 | 03 |  | Negative | Negative |  | Negative | Positive |  |  |

NR: Nonreactive; ND: Not done; F: female; M: male.
